# Cloning and sequencing *gB*, *gD*, and *gM* genes to perform the genetic variability of bovine herpesvirus-1 from Indonesia

**DOI:** 10.14202/vetworld.2018.1255-1261

**Published:** 2018-09-14

**Authors:** Dewi Noor Hidayati, Tri Untari, Michael Haryadi Wibowo, Koichi Akiyama, Widya Asmara

**Affiliations:** 1 Doctoral Program, Department of Microbiology, Faculty of Veterinary Medicine, Universitas Gadjah Mada, Jl. Fauna No. 2, Caturtunggal, Depok, Karangmalang, Sleman, Daerah Istimewa Yogyakarta 55281, Indonesia; 2 PUSVETMA (Pusat Veteriner Farma), The Ministry of Agriculture of The Republic of Indonesia, Jl. Frontage Ahmad Yani Siwalankerto No. 68-70, Ketintang, Gayungan, Surabaya, Jawa Timur 60231, Indonesia; 3 Department of Microbiology, Faculty of Veterinary Medicine, Universitas Gadjah Mada, Jl. Fauna No. 2, Caturtunggal, Depok, Karangmalang, Sleman, Daerah Istimewa Yogyakarta 55281, Indonesia; 4 Advanced Research Support Centre (ADRES), Ehime University, 3-5-7 Tarumi, Matsuyama, Ehime 790-8566, Japan

**Keywords:** bovine herpesvirus-1.1, bovine herpesvirus-1.2, *glycoprotein B*, *glycoprotein D*, *glycoprotein M*

## Abstract

**Aim:**

Previous research has shown that bovine herpesvirus-1 (BHV-1) in Indonesia was closely related to subtype-1 based on *glycoprotein D* genes. This study aimed to analyze the genetic variability of the BHV-1 isolated from the recent case in Indonesia not only based on *gD* but also other genes such as *gB* and *gM* and to study the homology and similarity of the sample to other BHV-1 isolated in other countries or regions.

**Materials and Methods:**

Samples were drawn from the tracheal organ in recent field case and prepared for DNA extraction. The *gB*, *gD*, and *gM* were amplified using nested polymerase chain reaction (nPCR) with our specifically designed primer pair and based on the specified bands of 350 bp *gB*, 325 bp *gD*, and 734 bp *gM* confirmed as BHV-1. The PCR product was ligated into pGEM-T and transformed into competent *Escherichia coli*. The purified plasmid was subsequently sequenced.

**Results:**

The virus sample isolated from the recent field case of infectious bovine rhinotracheitis (IBR) from Indonesia showed variability based on the *gB*, *gD*, and *gM* sequences. However, all of the genes had high similarity (98-100%) to BHV-1.2.

**Conclusion:**

The recent field case of IBR in Indonesia was similar to BHV-1.2.

## Introduction

The genome *Bovine herpesvirus-1* (BHV-1) consisted of two segments, which were unique long (UL) and unique short (US) [1]. The genome herpesvirus is large, double-stranded, and linear [2]. The BHV-1 included in class D Herpesvirus [3]. The US located between internal repeat and terminal repeat and had two alternative directions toward the UL. Inversely, the UL was fixed orientation [4,5]. Each segment encoded some glycoproteins, such as *gB, gD*, and *gM*. The glycoproteins played an important role in virus-cell interaction [6]. Glycoproteins B, D, and M were identified in the virion [7]. The glycoproteins B and D have been extensively studied [8]. The glycoprotein D played an important role in virus cell as binding protein receptors [9,10]. The glycoprotein B had significant variability used in describing molecular evolutionary [11,12]. Glycoprotein M was the only conserved glycoprotein among Herpesviridae [13-15]. The glycoprotein M was inessential for lytic replication [16] and increased by dexamethasone treatment [17].

The BHV-1 has been divided into three subtypes, which were BHV-1.1, BHV-1.2a, and BHV-1.2b [18]. BHV-5 used to be classified as BHV-1.3. There were several methods that have been used to differentiate the subtypes, such as a monoclonal antibody of specific antigen and DNA fingerprinting by visualizing the presence of the restriction site [19-21]. Recently, sequencing of the whole genome was necessary to differentiate the BHV-1 field strain from vaccine strain based on SNPs [22,23]. Some BHV-1 strains such as Cooper strain had the pattern of genome mapping that used restriction enzyme [24]. The *gC* mapping could differentiate the BHV-1 from BHV-5, which had different patterns in the site of restriction enzyme [25]. There was a restriction fragment length polymorphism that could differentiate BHV-1.1, BHV-1.2, and BHV-5 [26]. The homology of the BHV-1.1 and BHV-1.2 was about 95% [20], and that of the *gD* of the BHV-1.1 and the BHV-1.2 was 98.1% [27] Meanwhile, the similarity of the BHV-1 and BHV-5 was 82% [9,10,28]. Recombination in alpha herpesviruses was an important evolution mechanism [21,29]. Molecular recombination frequently occurred during the replication cycle of the BHV-1 [30]. Recombination might modify the virulence of alpha herpesvirus [29] and gave different isomeric forms of the virus [31]. The recombination could occur both *in vivo* and *in vitro* [32]. Some studies have examined the function of recombinant viruses with a mutation such as deletions or replacements of individual genes in the developing vaccines [33-35].

This study aimed to perform to test of the characteristic of BHV-1 found in the field case of infectious bovine rhinotracheitis (IBR) disease and to study the homology and similarity of the samples to other BHV-1 isolated in other countries of the region using some genes both from UL and US segments.

## Materials and Methods

### Ethical approval

This study was approved by The Committee for Safe Handling of Living Modified Organism in Ehime University (Permission number: H28-05) and carried out according to the guidelines of the committee. The sample was obtained from The Animal Disease Investigation Centre (ADIC), Lampung based on the letter of approval for material transfer No. 05010/PD.650/F5.H/06/2015.

### Materials

The samples of the study were drawn from the tracheal section in the field cases. They were obtained from the Animal Disease Investigation Center, Lampung, of the Ministry of Agriculture of the Republic of Indonesia.

### The preparation of samples

The tracheal samples were washed using phosphate-buffered saline (PBS) 3 times. The mixture of 1000 IU/ml Penicillin (Meiji Pharmaceutical Industries, Indonesia), 1000 µg/ml Streptomycin-sulfate Penicillin (Meiji Pharmaceutical Industries, Indonesia), 250 µg/ml Kanamycin Penicillin (Meiji Pharmaceutical Industries, Indonesia), and the PBS was used in the first washing. And then, they were washed 3 times using pure PBS. Subsequently, they were crashed out using DMEM and quartz sand and centrifuged at 1000 rpm for 5 min. The suspension was filtered using Sartorius Filter 0.2 mµ. The filtered fluid was prepared for DNA extraction.

Viral DNA was extracted using QIAamp DSP DNA Mini Kit (Qiagen). The DNA eluted with 200 µl AE Buffer that contained 10 mM Tris-Cl and 0.5 mM EDTA pH 9.0. The quality of DNA extraction was measured using BioSpec-nano spectrophotometer (Shimadzu Biotech, Japan).

### Mapping and primer designing for *gB*, *gM*, and *gD* gene amplification

**Figure-1:**
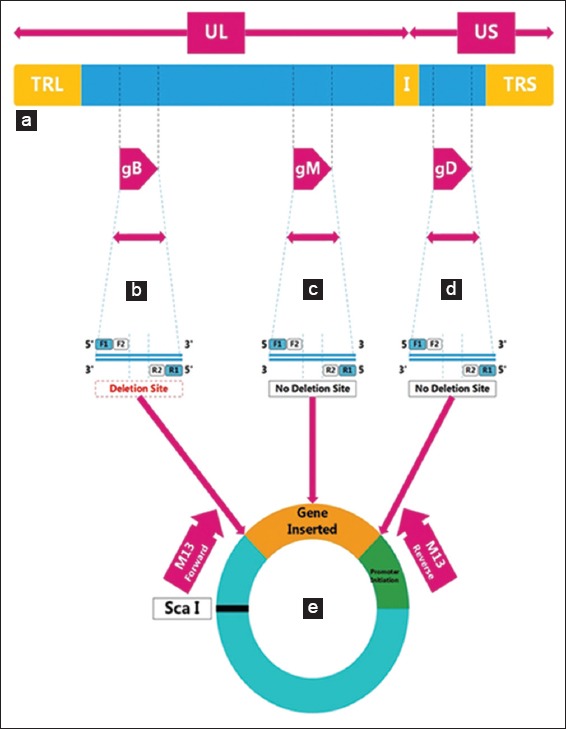
(a) The genome organization of *Bovine herpesvirus-1*, (b) the amplification of *gB* using two pairs of primers, (c) the amplification of *gM*, (d) the amplification of *gD*, and (e) the gene was cloned into pGEM-T.

We designed two pairs of primer to amplify *gB*, *gD*, and *gM* in examining the variability of Indonesian BHV-1 sample. The sequence of these primers is shown in Table-1 [36]. Mapping of the amplified fragment within the whole genome of BHV-1 is shown in Figure-1.

**Table-1:** The primers used in this study.

Names of primers	Sequences	Positions in the genome (Ref.KU198480.1)	PCR product lengths	Source
*gB*-1F	5’GACGTGTTCTCGCTGCTCTAC 3’	55807-55827	350 bp	Present study
*gB*-1R	5’TACGTGCTGCCCGCCCA 3.’	56299-56283		
*gB*-2F	5’CCTGCTATGGGCGACGTGG 3’	55880-55898		
*gB*-2R	5’GCGCGATGTTCTCCTTGTAAATG 3’	56229-56207		
*gD*-1F	5′GCTGTGGGAAGC GGTACG-′3	118086-118103	325 bp	Wiedmann *et al.*[36]
*gD*-1R	5′GTCGACTATGGCCTTGTGTGC-′3	118551-118531		
*gD*-2F	5’ACGGTCATATGGTACAAGATCGAGAGCG‘3	118129-118156		
*gD*-2R	5’CCAAAGGTGTACCCGCGAGCC‘3	118451-118451		
*gM*-1F	5’GTATCATATGAACGCGCTGGC 3’	85854-85834	734 bp	Present study
*gM*-1R	5’CTTAGCGGTATGGTTGGCG 3’	84977-84995		
*gM*-2F	5’TGCTGTCCCACAAGATCATGGTCT 3’	85759-85736		
*gM*-2R	5’CTCCTCGTCGTCAGAGGCGAC 3’	85026-85046		

PCR=Polymerase chain reactions

### Alignment, amplification, and cloning the targeted gene

The study intended to identify the characteristics of Indonesian BHV-1 samples based on *gB*, *gM*, and *gD*. The alignment was determined using DNAStar program with eight (n=8) reference genes obtained from GenBank with accession numbers (AJ004801.1, KU198480.1, JX898220.1, KM258881.1, KM258880.1, KM258882.1, KM258883.1, and U.146561). The alignment of *gB* showed the deletion site of about 18 nucleotides (nt), which determined the BHV-1.2 strain B589. The BHV-1.1 and other strain of BHV-1.2 did not have any deletion site in this sequence. The alignment of *gD* showed 29 points of mutation of the sequence to differentiate between BHV-1 and BHV-5, and the alignment of *gM* showed 3 points mutation (at position of 507, 679, and 850), which could differentiate between BHV-1.1 and BHV-1.2.

The polymerase chain reaction (PCR) assay proceeded in two rounds. Each round consisted of 2.5 mM MgCl_2_, 0.3 mM for each of dNTPs, 1 U of DNA Polymerase, 10 pmol forward and reverse primer, and template DNA of <100 ng per reaction until total volume was up to 50 µl for PCR grade water (KAPA HiFi HotStart ReadyMix, KAPABiosystem).

The optimization of the PCR of *gB* was performed as follows: Initial denaturation for 3 min at 95°C, denaturation for 20 s at 98°C, annealing for 15 s at 53°C which were run in 35 cycles, while the extension lasted for 15 s at 72°C and the final extension lasted for 5 min at 72°C. The second round of the amplification of the *gB* was performed as follows: Initial denaturation for 3 min at 95°C, denaturation for 20 s at 98°C, annealing for 15 s at 59.5°C which were run in 35 cycles, while the extension lasted for 10 s at 72°C and the final extension lasted for 5 min at 72°C. The optimization of the PCR of *gM* was performed as follows: Initial denaturation for 3 min at 95°C, the denaturation for 20 s at 98°C, annealing for 30 s at 55°C which were run in 37 cycles, while the extension lasted for 30 s at 72°C and final extension lasted for 5 min at 72°C. The second round of the amplification the *gM* was performed as follows: Initial denaturation for 3 min at 95°C, denaturation for 20 s at 98°C, annealing for 15 s at 58°C which were run in 37 cycles, while the extension lasted for 10 s at 72°C and the final extension lasted for 5 min at 72°C. The optimization of the PCR of *gD* was performed as follows: Initial denaturation for 3 min at 95°C, denaturation for 20 s at 98°C, annealing for 15 s at 53°C which were run in 35 cycles, while the extension lasted for 15 s at 72°C and the final extension lasted for 5 min at 72°C. The second round of the amplification of *gD* was performed as follows: Initial denaturation for 3 min at 95°C, denaturation for 20 s at 98°C, annealing for 15 s at 59.5°C which were run in 35 cycles, while the extension lasted for 10 s at 72°C and the final extension lasted for 5 min at 72°C.

The PCR product of the second round was ligated into plasmid pGEM^®^-T (Promega) using T4 ligation enzyme (Promega) once it has been purified in FastGene purification (NIPPON Genetics) and attached using poly A at the 3’-end with 10x A-attachment (TOYOBO). The ligated plasmid was transformed into competent *Escherichia coli* C3040 (NEB^®^stable, High efficiency) and kept in LB agar overnight at 37°C. The single white colony was picked up and transferred into LB broth media containing 100 µl ampicillin per mL and incubated at 37°C with constant shaking overnight. The recombinant cloned plasmid was prepared to release the DNA fragment by chemistry lysis [37]. Moreover, it was confirmed by gel agarose, and any appropriate cloned plasmid was subsequently subjected to PCR amplification using the designated primers. Only the positive inserted cloned plasmid was subsequently subjected to PCR sequencing.

### The establishment of cloned plasmid and nt sequencing

The cloned plasmid was purified using FastGene (NIPPON Genetics) and subjected to sequencing with Sanger methods. The cycling of the forward and reverse PCR sequencing was based on the protocol of BigDye Terminator v3.1 Cycle Sequencing Kit at the annealing temperature of 50°C using M13. The Sanger sequencing was run using 3130 Genetic Analyzer (Applied Biosystems). The data were analyzed using DNAStar Lasergene software. The resulting consensual sequence of the forward and reverse sequence was submitted and compared to the reference genes retrieved from GenBank with accession numbers (AJ004801.1, KU198480.1, JX898220.1, KM258881.1, KM258880.1, KM258882.1, KM258883.1, and U.146561). Subsequently, BLAST software (https://blast.ncbi.nlm.nih.gov) was used to confirm the similarity of the sample to the alpha herpesvirus. nt sequences were deposited in the DNA Data Bank of Japan with accession numbers (LC349880, LC349881, and LC349882).

### Sequence alignment

Sequence results were analyzed using SeqMan (DNAStar Lasergene, version 7). All of the assembled sequences were aligned using Clustal W method packaged in MegAlign software (DNAStar Lasergene, version 7) based on multiple sequence alignments [38,39].

## Results

### The sequence of *gD* between BHV-1 and BHV-5

The *glycoprotein D* gene of Indonesian sample was sequenced, which covered 323 bp. This segment has been used for the detection and monitoring of IBR diseases in Indonesia. We analyzed the results and aligned them with the reference genes of BHV-1.1 (n=3) (Accession numbers: AJ004801.1, KU198480.1, and JX898220.1), BHV-1.2 (n=4) (Accession numbers: KM258880.1, KM258881.1, KM258882.1, and KM258883.1), and BHV-5 (n=1) (Accession number: U.1465610). The sequence of BHV-1.1 was in *gD* reference position of 118129-118449, BHV-1.2=118905-119225, and BHV-5=559-878 using Clustal Omega (MegAlign software, DNAStar Lasergene). The substitution mutation was observed within the sequence (Table-2). SNPs occurred in 29 points between BHV-1 and BHV-5. Transversion occurred in 6 points. The transition occurred in 23 points. The base mutation between adenine and guanine occurred in almost the same number of points as the base mutation between cytosine and thymine. The previous study used *gD* to examine the difference between BHV-1 and BHV-5. The similarity between the samples and reference genes was identical with BHV-1.2 strain B589 (99.7%). The samples had about 92.3% similarity to BHV-5 and 98.8-99.1% similarity to other BHV-1.1. The similarity between BHV-1 and BHV-5 was about 92.0-92.9%.

**Table-2:** The alignment results of the *gD* of BHV-1.1, BHV-1.2, and BHV-5. There were 29 SNPs. Transition occurred at nt 330, 386, 396, 417, 426, 433, 439, 462, 477, 481, 483, 506, 522, 544, 555, 558, 561, 570, 592, 603, 608, 612, and 321. Transversion occurred at nt 371, 381, 555, 568, and 322.

Name of sequence	3	3	3	3	3	4	4	4	4	4	4	4	4	5	5	5	5	5	5	5	5	5	6	6	6	6	6	3	3

	3	7	8	8	9	1	2	3	3	6	7	8	8	0	2	4	5	5	6	6	7	9	0	0	1	1	1	2	2

	0	1	1	6	6	7	6	3	9	2	7	1	3	6	2	4	5	8	1	8	0	2	3	8	2	4	5	1	2
**Alignment sequence of *gD***	**Position of the nucleotide of BHV-1**																												

BHV-1.1 complete (AJ004801.1)	C	C	G	G	T	A	T	A	C	G	A	A	T	T	A	A	T	A	T	G	T	T	A	G	G	C	T	T	G
BHV-1.1 strain cooper (KU198480.1)	•	•	•	•	•	•	T	•	C	G	•	•	•	•	•	•	•	•	•	•	•	T	A	G	G	•	•	•	•
BHV-1.1 isolate NVSL (JX898220.1)	•	•	•	•	•	•	T	•	C	G	•	•	•	•	•	•	•	•	•	•	•	T	A	G	G	•	•	•	•
BHV-1.2 strain K22 (KM258880.1)	•	•	•	•	•	•	C	•	C	A	•	•	•	•	•	•	•	•	•	•	•	C	A	G	G	•	•	T	A
BHV-1.2 strain SM 02 (KM258882.1)	•	•	•	•	•	•	T	•	T	A	•	•	•	•	•	•	•	•	•	•	•	T	G	A	G	•	•	•	•
BHV-1.2 strain Sp 177 (KM258883.1)	•	•	•	•	•	•	T	•	T	A	•	•	•	•	•	•	•	•	•	•	•	T	G	A	G	•	•	•	•
BHV-1.2 strain B589 (KM258881.1)	•	•	•	•	•	•	T	•	C	A	•	•	•	•	•	•	•	•	•	•	•	T	G	A	A	•	•	•	•
Gen D_L/9_320bp	•	•	•	•	•	•	T	•	C	A	•	•	•	•	•	•	•	•	•	•	•	T	G	A	A	•	•	T	G
BHV-5 (U146561)	T	G	C	A	C	G	C	G	C	G	G	G	C	C	G	G	C	G	C	C	C	T	G	G	G	A	G	C	C

	**Position of nt of BHV-5**																												

	5	6	6	6	6	6	6	6	6	7	7	7	7	7	7	7	8	8	8	8	8	8	8	8	8	8	8	8	8
	7	2	3	3	4	6	7	8	8	1	2	3	3	5	7	9	0	0	1	1	1	4	5	5	6	6	6	7	8
	9	0	0	5	5	6	5	2	8	0	6	0	2	5	1	3	4	7	0	7	9	1	2	7	1	3	4	9	0

BHV=*Bovine herpesvirus*, SNP=Single nucleotide polymorphisms, nt=nucleotides

### The sequence of *gB* between BHV-1.1 and BHV-1.2

The sequences of *glycoprotein B* gene of the sample (L/9) covered 335 bp nt. The alignment was made between the sample L/9 and the reference genes of BHV-1.1 (n=3) and BHV-1.2 (n=4) using Clustal W (MegAlign software, DNAStar Lasergene). The results showed that the BHV-1.1 *gB* reference position was 55924-56227, while the BHV-1.2 reference position was 55652-55955. These sequences resulted in the deletion and substitution as summarized in Table-3. There were 18 nt deletions occurring in the sequences between BHV-1.1 and BHV-1.2 strain B589 at nt 259-276. The substitution (the transitions of adenine to guanine and thymine to cytosine) was observed at the sequence in the positions 304, 321, and 438. The samples L/9 did not have any deletion. The sample L/9 showed about 99.1% similarity with BHV-1.1 and 99.7-100% similarity with BHV-1.2. It showed that the sample L/9 was closely related to BHV-1.2.

**Table-3:** The alignment results of *gB* between BHV-1.1 and BHV-1.2 There were 18 nt deletions except the sequence of BHV-1.2 strain B589. The sample L/9 did not have any deletion site. There were four single nt polymorphisms occurring at 251, 304, 321, and 438. The point of mutation was substitution transition (A to G and T to C) covered with blue box that differentiated BHV-1.1 and BHV-1.2.

Name of sequence	2	‒	‒	‒	‒	‒	‒	‒	‒	‒	‒	‒	‒	‒	‒	‒	‒	‒	‒	3	3	4

	5	‒	‒	‒	‒	‒	‒	‒	‒	‒	‒	‒	‒	‒	‒	‒	‒	‒	‒	0	2	3

	1	‒	‒	‒	‒	‒	‒	‒	‒	‒	‒	‒	‒	‒	‒	‒	‒	‒	‒	4	1	8
**Alignment sequence *gB***	**Position of nt of BHV-1.1 and BHV-1.2**																					

BHV-1.1 complete (AJ004801.1)	C	‒	‒	‒	‒	‒	‒	‒	‒	‒	‒	‒	‒	‒	‒	‒	‒	‒	‒	A	A	T
BHV-1.1 strain cooper (KU198480.1)	C	‒	‒	‒	‒	‒	‒	‒	‒	‒	‒	‒	‒	‒	‒	‒	‒	‒	‒	A	A	T
BHV-1.1 isolate NVSL (JX898220.1)	C	‒	‒	‒	‒	‒	‒	‒	‒	‒	‒	‒	‒	‒	‒	‒	‒	‒	‒	A	A	T
BHV-1.2 strain K22 (|KM258880.1)	T	‒	‒	‒	‒	‒	‒	‒	‒	‒	‒	‒	‒	‒	‒	‒	‒	‒	‒	G	G	C
BHV-1.2 strain SM 023 (KM258882.1)	C	‒	‒	‒	‒	‒	‒	‒	‒	‒	‒	‒	‒	‒	‒	‒	‒	‒	‒	G	G	C
BHV-1.2 strain Sp 1777 (KM258883.1)	C	‒	‒	‒	‒	‒	‒	‒	‒	‒	‒	‒	‒	‒	‒	‒	‒	‒	‒	G	G	C
BHV-1.2 strain B589 (KM258881.1)	C	G	C	C	G	C	G	A	G	C	C	C	G	G	C	G	C	C	C	G	G	C
Gene B_L/9	C	‒	‒	‒	‒	‒	‒	‒	‒	‒	‒	‒	‒	‒	‒	‒	‒	‒	‒	G	G	C

	**Position of nt of BHV-1.2 strain B589**																					

	2	2	2	2	2	2	2	2	2	2	2	2	2	2	2	2	2	2	2	3	3	4
	5	5	6	6	6	6	6	6	6	6	6	6	7	7	7	7	7	7	7	2	3	5
	1	9	0	1	2	3	4	5	6	7	8	9	0	1	2	3	4	5	6	2	9	6

BHV=*Bovine herpesvirus*, A to G=Adenine to guanine, T to C=Thymine to cytosine, nt=Nucleotides

### The sequence of *gM* between BHV-1.1 and BHV-1.2

The sample (L/9) covered 689 bp of *gM* segment. The alignment was made between the sample L/9 and the reference genes of BHV-1.1 (n=3) and BHV-1.2 (n=4) using Clustal W (MegAlign software, DNAStar Lasergene, USA). The results showed that the BHV-1.1 *gM* reference position was 85089-85735, while the BHV-1.2 reference position was 84874-85520. The sequences resulted in the substitution mutation as summarized in Table-4. There were three mutation sites found in the sequences between BHV-1.1 and BHV-1.2. The substitution mutation that differentiated the BHV-1.1 and the BHV-1.2 was observed at the sequence in the positions 507, 678, 849, and 1146. The majority of the mutation points were found between thymine and cytosine (3 points). The samples of L/9 thymine were at nt 777, which were different from all BHV-1. There were four points indicative of the deletion of one nt occurring between BHV-1.1 AJ004801 and other BHV-1 at nt 505, 517, 937, and 951. The similarity percentage between the sample L/9 and BHV-1.1 was about 99.3% and that between the sample L/9 and BHV-1.2 was 99.4-99.9%. It showed that the sample L/9 closely related to BHV-1.2.

**Table-4:** The alignment results of the *gM* of BHV-1.1 and BHV-1.2. There were nine SNPs at nt 492, 507, 678, 777, 849, 897, 900, 901, and 1146. The blue box showed the location of SNPs which could differentiate BHV-1.1 and BHV-1.2. The red box showed the mutation of sample L/9. Only transition occurred in the sequence.

Name of sequence	4	5	5	5	6	7	8	8	9	9	9	9	1

	9	0	0	1	7	7	4	9	0	0	3	5	1

	2	5	7	7	8	7	9	7	0	1	7	1	4

													6
**Alignment sequence of *gM***	**Position of nucleotide of BHV-1.1**												

BHV-1.1 complete (AJ004801.1)	C	–	T	G	T	C	T	C	C	C	C	–	A
BHV-1.1 strain cooper (KU198480.1)	C	G	T	–	T	C	T	C	C	C	–	C	A
BHV-1.1 isolate NVSL (JX898220.1)	C	•	T	–	T	C	T	C	C	C	–	C	A
BHV-1.2 strain K22 (|KM258880.1)	C	•	C	–	C	C	C	T	T	T	–	C	G
BHV-1.2 strain SM 023 (KM258882.1)	C	•	C	–	C	C	C	C	C	C	–	C	G
BHV-1.2 strain Sp 1777 (KM258883.1)	C	•	C	–	C	C	C	C	C	C	–	C	G
BHV-1.2 strain B589 (KM258881.1)	A	•	C	–	C	C	C	C	C	C	–	C	G
gM L/9	C	•	C	–	C	T	C	C	C	C	–	C	G

	**Position of nucleotide of BHV-1.2**												

	4	5	5	5	6	7	8	8	9	9	9	9	1
	9	0	0	1	7	7	4	9	0	0	3	5	1
	2	5	7	7	8	7	9	7	0	1	7	2	4
													6

BHV=*Bovine herpesvirus*, SNP=Single nucleotide polymorphisms

## Discussion

The first case of IBR in Indonesia was in 1981. The predicted causal factor was due to live cattle importation [40,41]. Previous research reported that only BHV-1.1 was found in the field case [42]. The case of IBR in Indonesia escalated. This study was a molecular study of the recent field case of IBR. The molecular analysis was based on sequencing the analysis of *glycoprotein B*, *glycoprotein D*, and *glycoprotein M* genes. The sequences of *gD*, *gB*, and *gM* were obtained by cloning the fragment into pGEM-T. The sequencing results of both forward and reverse sequencings using M13 primer were analyzed. Based on the alignment of *gD* 323 bp, the sequence contained transition and transversion in 29 points. Considering the fragment, the sample L/9 was similar to BHV-1.2 strain B589 (99.7%). However, many studies that also used *gD* as a marker gene to differentiate BHV-1 and BHV-5 [8], particularly amino acid in US segment of BHV-5, had 69-98% similarity to BHV-1 [28]. The similarity of the sample (L/9) to the reference gene of BHV-1 was >98.1-99.7%, and that to the reference gene of BHV-5 was >92%. Specifically, the similarity of the sample (L/9) to BHV-1.1 was >98%, and that to BHV-1.2 was 99%. The sample L/9 was significantly different from BHV-5 and closely similar to BHV-1.2.

The alignment result of *gB* contained a deletion of 18 nt (259-276). The deletion site could differentiate BHV-1.2 strain B589 from other BHV-1. The sample L/9 had a closer similarity to BHV-1.2 (>99.0%) than BHV-1.1(>98%). The fragment could be a good marker based on the molecular weight of the segment. The fragment could differentiate BHV-1.2, particularly BHV-1.2 strain B589.

Based on the alignment of *gM*, the sequence contained substitution (only transition). The *gM* served the function as secondary envelopment and formed with *gN* that inhibited transporter-associated processing [43]. The *gM* fragment was more conserved than *gB* and *gD* to measure the evolutionary molecular genetics. The sample L/9 had close similarity to BHV-1.2 strain SP 1777 (KM 258883.1) and BHV-1.2 strain SM 023 (KM 258882.1).

This molecular study showed that there was variability of the samples drawn from field case among the reference genes (BHV-1.1 and BHV-1.2 BHV-5). The use of *gB*, *gD*, and *gM* led to the differentiation of BHV-1.1 and BHV-1.2 and particularly BHV-1.2 strain B589.

## Conclusion

The sequence variability within *gD, gB, and gM* genes of BHV-1 isolated from Indonesian samples are mostly due to transition and transversion mutation. The result also indicated that the causative agent for recent IBR cases in Indonesia is related to BHV-1.2.

## Authors’ Contributions

DNH formulated the objectives of the study, designed, and planned; TU, MHW, KA, and WA supervised the experiments and corrected the manuscript. All of the authors read and approved the final manuscript.
